# Intubation in Laryngeal Tuberculosis

**Published:** 1892-04-02

**Authors:** 


### THE PRACTITIONER'S MIRROR AND RETROSPECT.

*The Editor will be glad to receive offers of co-operation and contributions from members of the profession. All letters should be addressed to* The Editor, The Lodge, Porchester Square, London, W.]

### MEDICINE.

### INTUBATION IN LARYNGEAL TUBERCULOSIS.

We do not often meet with cases of laryngeal tuberculosis with symptoms of obstructive dyspnœa, for the reason that long before the tubercular infiltration has occluded the glottic opening it has almost always undergone ulceration and is breaking down, the wasted and weak condition of the patient preventing the muscular exertion which, under normal conditions, would render the partial occlusion very distressing. Yet we are occasionally confronted with cases of laryngeal tuberculosis with stenosis, and the question arises as to the best mode of affording relief.

We believe that M. Massei was the first who intubated these cases, and his experience in three cases was most favourable, and seemed to show that stenosis may be got to yield in a surprisingly short space of time.

In a recent report by Dr. F. H. Hopkins to the Section in Laryngology and Rhinology, of the New York Academy of Medicine,* we are reminded that both Dr. Brown and Dr. D. C. Cox, of New York, have each performed intubation once for the relief of acute laryngeal obstruction from tuberculosis. We have ourselves had such an excellent result from intubation for the relief of acute syphilitic œdema that we can most readily believe that death was rendered less painful and distressing in inevitably fatal conditions such as Dr. Brown's and Dr. Bell's were. Dr. Hopkins himself reports a case, that of a woman in whom the procedure of intubation was apparently beneficial not only by relieving acute symptoms, but in removing, to a certain extent, the laryngeal obstruction itself. The case of Mrs. B., when she first came under observation, presented the following condition of the larynx: The mucous membrane in general was pale. The vocal bands were somewhat hyperæmic and thickened, and on phonation failed to approximate. The arytenoids and aryepiglottic folds were normal in appearance. At the posterior commissure, however, in the interarytenoid space there was distinct thickening, the mucous membrane being lobulated and decidedly pale in colour. Two wart-like masses could be seen projecting into the lumen of the larynx, no doubt sufficient to interfere with the action of the vocal bands. The ventricular bands were normal, and there was no ulceration present. The diagnosis of laryngeal tuberculosis was based upon the anæmia of the larynx, and the post-commissural infiltration. Just two years later she came again under treatment, and was then suffering from cough and hoarseness, but the larynx at that time also did not present the characteristic appearance of the tubercular process, although the thickening and infiltration referred to above had become more marked, and there was evidence of early pulmonary disease at the right apex. In about another two and a-half years —that is, about January of this year—from a gradual decline, the symptoms became marked in severity, the ulcerative process in the larynx having advanced so as to present the typical appearance of the disease. There was a steady encroachment on the lumen of the larynx, due to thickening and infiltration of all the surrounding parts, the interarytenoid thickening being especially noticeable. For three weeks preceding January 30th suffocative attacks came on nightly, and increased constantly in severity. There was also marked dyspnœa an exertion. Her general condition declined more rapidly. She had no appetite; was so weak that her visits to the hospital were serious drafts upon her strength. Her expression was one of painful anxiety, and it was evident to Dr. Hopkins that tracheotomy would soon become necessary because of the steady narrowing of the already dangerously narrow rima glottidis, and that operation was advised by his colleagues. He, however, resolved to try intubation instead, if it became necessary, as, in fact, it did very soon.

* *New York Med. Journ.*, Feb. 27th, 1892.

Examination of the larynx showed that the interarytenoid thickening had advanced to such an extent that the vocal cords were covered for fully a third of their length. The ventricular bands were much thickened, and appeared like firm fibrous masses, encroaching upon the rima glottidis upon either side.

After cocainising the pharynx and larynx, the attempt was made to insert the largest tube in the O'Dwyer case, viz., that for a child of twelve years of age. Three attempts were made to insert this tube, and all the force was employed that it seems prudent to use, but the tube failed to pass. Even the smaller size met with much resistance—a resistance due largely, in Dr. Hopkins' opinion, to muscular spasm. The presence of the tube excited violent paroxysms of coughing. After the tube had been in position some minutes, and after the cord attached to it had been removed, the coughing was less severe. Although the tube was so small, the patient breathed much easier than before its insertion.

The tube was expelled later during an attack of coughing. The dyspnœa, however, was relieved, and the patient passed a better night than for three weeks. For twenty-four hours following, the patient suffered considerable local pain, and swallowed with difficulty, although there had previously been no dysphagia. She also coughed more than usual, expectorating blood-stained mucus, this and the dysphagia being due to the traumatism of the intubation. The dyspnœa was relieved, because the force exerted upon the tube tore away a portion of the posterior commissural thickening, and doubtless the remainder subsided somewhat by contraction, and from relief to the engorgement from the bleeding. With the relief of the breathing the patient's appetite improved again, and then followed a gain in strength and general condition. Mrs. B. so far recovered her strength that she not only helped to take care of herself, but assisted in the care of her husband, who is confined to bed from pulmonary phthisis. Both patients are now dead, but Mrs. B. had no suffocative attacks after the intubation, and, in fact, during the last weeks of her life the lumen of arynx, enlarged by the destructive ulceration, was greater than it had been during any time in the preceding year.

Dr. Hopkins remarks that in the condition under consideration it is not unwarrantable to make the assertion that the operation ought always to be done instead of tracheotomy. The ease and quickness with which the operation may be done; the comparative absence of shock; the absence of a wound in the tissues predisposed to necrosis; the fact that air reaches the lungs through the natural passages; the possibility that after a few hours the tube may be dispensed with, in fact all the familiar arguments in favour of intubation appeal with great force for its use in these cases.

When it is a question as to whether tracheotomy or intubation shall be done we most entirely agree with Dr. Hopkins' position, but it is well to remember that if the case is not too hoplessly advanced curettement combined with the application of lactic acid often gives astonishingly good results, and may even produce cicatrisation of the affected larynx. Intubation can only hasten the ulcerative processes, and give relief to the obstructive dyspnœa at the same time. But where the services of a skilled laryngologist are unavailable intubation affords a comparatively easy method of removing the acute obstruction. It is the procedure which will prove more generally useful than any other, and Dr. Hopkins has done service in giving such a full account of his most interesting case from which we have quoted. It must not be supposed that intubation in any adult is easy of performance; it is more difficult than in children, and in a painful disease like laryngeal tuberculosis the procedure must be attended with increased difficulty.

